# Incidental Diagnosis of Situs Inversus Totalis in a 45‐Year‐Old Male Who Presented With Acute Asthma Exacerbation: A Case Report and Brief Literature Review

**DOI:** 10.1002/ccr3.71425

**Published:** 2025-11-05

**Authors:** Ragasa Getachew Bayisa, Lensa Million Baharu, Mohammed Mecha Abafogi, Yosef Bekele Bonsa, Mohammed Kedir Shukri, Tamirat Godebo Woyimo

**Affiliations:** ^1^ Department of Internal Medicine, Faculty of Medical Sciences, Institute of Health Jimma University Jimma Ethiopia; ^2^ Department of Clinical Radiology, Faculty of Medical Sciences, Institute of Health Jimma University Jimma Ethiopia; ^3^ Division of Cardiology Jimma University Jimma Ethiopia; ^4^ Department of Internal Medicine, Faculty of Medical Sciences, Institute of Health Arba Minch University Arba Minch Ethiopia

**Keywords:** asthma exacerbation, computed tomography, dextrocardia, incidental finding, radiology case report, situs inversus totalis

## Abstract

Incidental situs inversus totalis (SIT) requires thorough anatomical mapping to exclude associated syndromes (e.g., Kartagener) and congenital anomalies. Early identification, patient education on mirrored anatomy, and multidisciplinary coordination are essential to prevent iatrogenic errors during future interventions, even in asymptomatic adults.

**Abbreviations**
DNAH5: dynein axonemal heavy chain 5
DNAI1: dynein axonemal intermediate chain 1
ECG: electrocardiogram
PCD: primary ciliary dyskinesia
TTE: transthoracic echocardiogram

## 1 Introduction

SIT is a rare congenital mirror‐image reversal of thoracoabdominal organs—heart apex pointing to the right (dextrocardia)—affecting about 1 in 8000–25,000 individuals (≈0.01%) [1, 2]. Usually asymptomatic, it is most often discovered incidentally on routine chest radiography or ECG, which may show a right‐sided gastric bubble, inverted P‐waves in lead I, and reversed R‐wave progression—reinforcing the need for careful clinical interpretation [2, 3, 4]. Recognizing SIT early is critical—not only to correctly interpret ECG and imaging—but also to avoid potentially hazardous errors (e.g., misplaced incisions or reversed lead placement) during emergency or anesthetic procedures [4]. About 20%–25% of individuals with SIT have underlying primary ciliary dyskinesia (PCD); conversely, nearly half of PCD patients exhibit situs inversus (the classic Kartagener triad) [5]. The coexistence of ciliary dysfunction with SIT significantly increases the risk of recurrent respiratory infections and infertility in males [5, 6].

Embryologically, SIT arises from disrupted left–right axis determination, often due to autosomal‐recessive mutations (e.g., DNAH5, DNAI1) with ~25% familial recurrence [7, 8]. If Kartagener syndrome is suspected—marked by chronic sinusitis, bronchiectasis, and SIT—further nasal nitric oxide testing and PCD gene sequencing are recommended [5]. Isolated SIT often occurs without other anomalies and typically carries a benign prognosis. Up to 95% of non‐SIT‐related dextrocardia cases with other congenital heart defects (e.g., ventricular septal defect, tetralogy of Fallot, transposition of the great arteries) require careful echocardiographic assessment [9]. Coexisting heterotaxy syndromes—with asplenia or polysplenia—heighten the risk of bacterial sepsis and intestinal malrotation, making comprehensive abdominal imaging (ultrasound or CT) essential at diagnosis [10, 11, 12].

Undiagnosed SIT may lead to iatrogenic complications during emergent procedures—misplaced incisions or unfamiliar organ orientation—so early identification provides a critical window for multidisciplinary coordination and anticipatory guidance [12, 13, 14, 15].

The aim of this report is to describe an isolated presentation of asymptomatic SIT in a middle‐aged male who presented with an acute asthma exacerbation. By reviewing similar African case reports we highlight the need for heightened clinical suspicion, ongoing follow‐up, and tailored management strategies in patients with incidental SIT.

## 2 Case History/Examination

A 45‐year‐old Ethiopian man, historically undiagnosed but clinically suspected of having asthma, presented after experiencing a progressive five‐day history of dyspnea, nocturnal dry cough, chest tightness, and a low‐grade fever of 37.7°C. He reported that over the years he had intermittently used inhaled medication purchased from a local pharmacy, especially when his breathing worsened, but had never received formal diagnostic testing. Symptoms worsened following a visit to a remote local health center, prompting his referral for advanced care. Despite adherence to short‐acting beta‐agonist (SABA) and low‐dose inhaled corticosteroids (ICS), nothing effectively controlled his condition. He denied any personal or family history of diabetes, hypertension, cardiac disease, hearing loss, neonatal complications, chronic productive cough, or recurrent infections. Married for 25 years with six healthy siblings, his background offered no additional clues.

On admission, vital signs were: blood pressure 112/70 mmHg, pulse 104 bpm, respiratory rate 26/min, and oxygen saturation 90% on room air. Physical examination revealed the use of accessory muscles of respiration and diffuse expiratory wheezes. Remarkably, his apical impulse was palpable in the right hemithorax; there was no nasal congestion or ear discharge. He was experiencing an acute moderate asthma exacerbation without signs of pneumonia. Other systemic examinations were unremarkable. After stabilization on day 6 of symptom onset, a chest radiograph unveiled mirror‐image findings: dextrocardia, a right‐sided aortic arch, and a gastric air bubble beneath the right hemidiaphragm.

**FIGURE 1:**
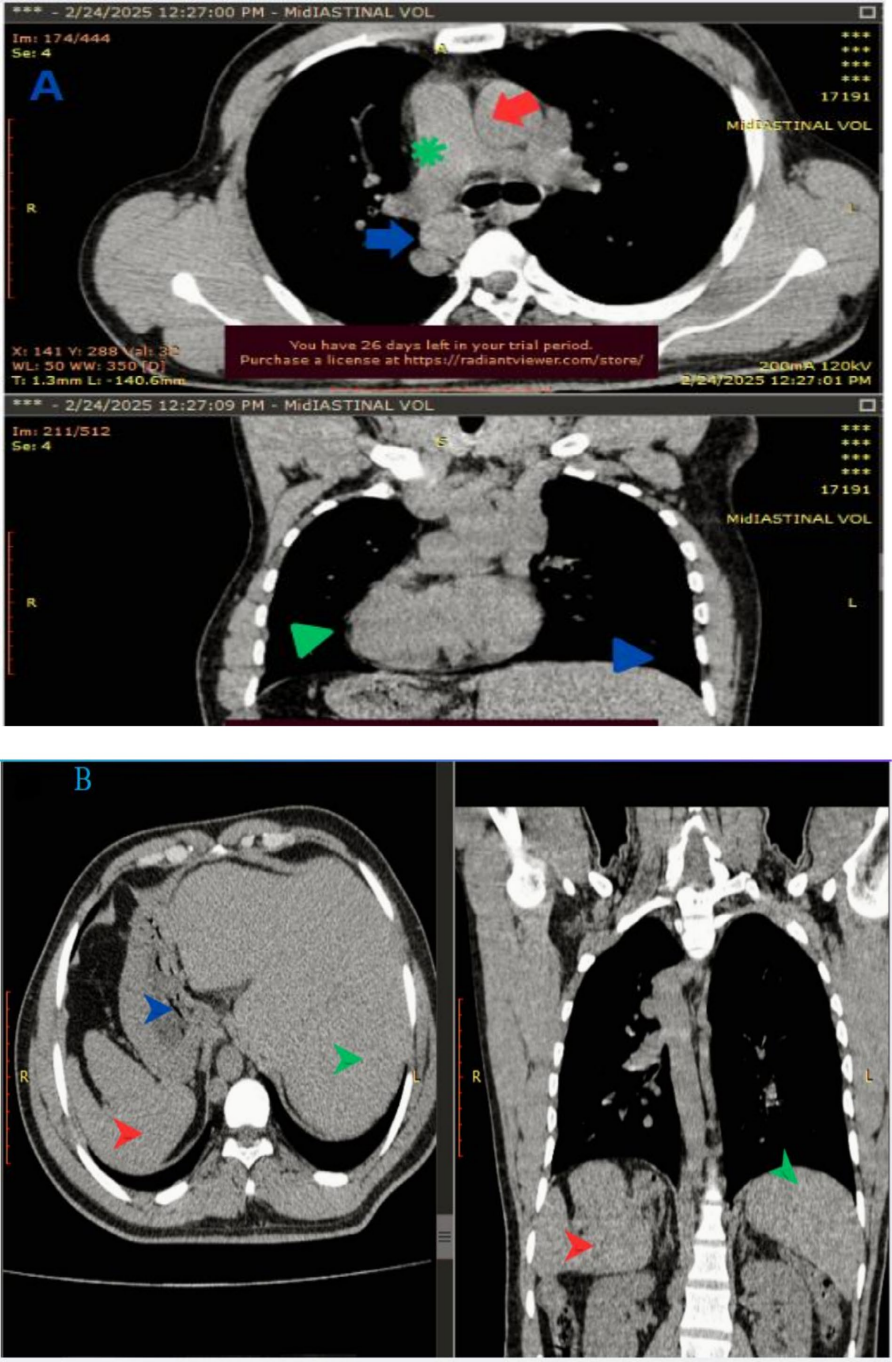
CT scan of the chest shows: (A) Pulmonary trunk (green asterisk) to the right, ascending aorta (red arrow) to the left and descending aorta to the right (blue arrow), the apex of the heart pointing to the right (green arrowhead). (B) Spleen (red arrow head), stomach (blue arrow head) and aorta visualized on the right side and liver (green arrow head) the left side.

Because of these mirrored findings, he was informed about the possibility of SIT and offered a complimentary pre‐contrast CT scan of the chest, abdomen, and pelvis, TTE, and ECG to which he consented. The CT corroborated complete visceral inversion—trilobed left lung, bilobed right lung; heart and great vessels mirrored; liver and gallbladder on the left; stomach, spleen, and colonic splenic flexure on the right (Figure 1A,B).

The scan showed no bronchiectasis (a noted artifact was confirmed not to represent pathology). An echocardiogram confirmed dextrocardia in the absence of structural abnormalities. A concurrent electrocardiogram (ECG) demonstrated characteristic features of dextrocardia, including sinus rhythm at 102 bpm, right axis deviation, inverted P waves in lead I, and absent R‐wave progression. These studies were performed on the patient's second day of admission, corresponding to the sixth day of symptom onset [24/02/2024].

Laboratory investigations revealed a normal leukocyte count (4900/μL) with a neutrophil predominance (71.6%). Liver function tests showed a mild elevation of alanine transaminase (ALT, 42.3 IU/L) with a normal aspartate transaminase (AST, 35.36 IU/L). The rest of the serologic workup was unremarkable, including normal renal function, electrolytes, and thyroid‐stimulating hormone (TSH). Inflammatory markers such as the erythrocyte sedimentation rate (ESR) were within normal limits. Serology for human immunodeficiency virus (HIV), antinuclear antibody (ANA), Venereal Disease Research Laboratory (VDRL), and hepatitis B and C was non‐reactive. Urinalysis was unremarkable.

The absence of chronic sinusitis, bronchiectasis, otorrhea, infertility indicators, or ciliary dysfunction rendered Kartagener syndrome unlikely. The final diagnosis comprised SIT with isolated late‐onset asthma. Kartagener syndrome was excluded, given the lack of its classical triad of chronic sinusitis, bronchiectasis, and ciliary dysfunction.

## 3 Interventions and Treatment

Acute management included supplemental oxygen, bronchodilators, and corticosteroids, which resulted in symptom resolution within 48 h.

## 4 Outcome and Follow‐Up

For long‐term care, the patient was advised to undergo annual spirometry to monitor his asthma, and was educated on how the presence of SIT might affect future medical or surgical procedures.

## 5 Discussion

SIT is rare (approx. 1 in 8000–25,000 live births), typically occurring sporadically without gender bias [1, 2]. Aside from genetic causes such as autosomal‐recessive inheritance (familial recurrence ~25%), some evidence suggests maternal diabetes or toxin exposure, although evidence remains limited [7, 8]. Our 45‐year‐old patient had no relevant family history or prenatal risk factors, consistent with most isolated SIT cases. His diagnosis was incidental, discovered after chest radiography conducted for asthma exacerbation—a presentation consistent with literature highlighting that SIT is often identified during evaluations for unrelated conditions.

It can be isolated or associated with multisystem congenital anomalies. Cardiac anomalies occur in approximately 5%–10% of SIT cases [9], and PCD features—neonatal respiratory distress (> 80%) and chronic wet cough (≈100%)—are classic; however, none were evident, ruling out Kartagener syndrome [5]. Gastrointestinal anomalies include intestinal malrotation (risk of volvulus) and biliary atresia, while heterotaxy may cause asplenia or polysplenia, increasing infection susceptibility [9, 10]. In our patient, echocardiography and CT revealed no structural heart disease, bronchiectasis, or malrotation, and abdominal imaging confirmed normal splenic morphology. His absence of recurrent infections, infertility, or neonatal distress further excluded common extra‐cardiac associations.

PCD and Kartagener syndrome were definitively ruled out in this case. PCD requires hallmark features: neonatal respiratory distress (> 80% of cases), chronic wet cough (95–100%), and recurrent otitis media or sinusitis [5, 6]. Our patient's only respiratory symptom was adult‐onset asthma, with no history of childhood infections or bronchiectasis on CT. Kartagener syndrome specifically necessitates the triad of SIT, chronic sinusitis, and bronchiectasis—none of which were present [5]. Nasal nitric oxide (nNO) testing, if performed, would likely have been normal (> 77 nL/min), contrasting with the markedly low levels in PCD. Genetic testing for PCD‐associated genes (e.g., DNAH5, DNAI1) was unwarranted given the lack of clinical indicators, reinforcing that his SIT was isolated [5, 8].

Comprehensive imaging—including chest X‐ray (suggesting dextrocardia), abdominal ultrasound (confirming visceral inversion), echocardiography (excluding structural heart defects), and CT (definitively mapping thoracoabdominal anatomy)—is essential in SIT [9, 10]. In our patient, CT confirmed isolated SIT with no additional anomalies, supported by classic ECG findings of dextrocardia (right‐axis deviation, inverted P waves in lead I, and absent R‐wave progression). Crucially, laevocardia (heart on the left despite reversed abdominopelvic organs) was excluded, as this carries a 95% risk of severe cardiac anomalies [9].

Based on our literature review using English‐word published articles, all known African‐reported cases of situs inversus totalis—from the first in 2003 to the most recent in April 2025—are summarized in Table S1. These reports span adolescents to elderly individuals, with many diagnoses occurring late: for example, a Nigerian woman was diagnosed at age 69 and a Ghanaian man at 59. Common presentations were respiratory: a 17‐year‐old Ethiopian boy with pneumonia was found to have Kartagener syndrome, and adults (e.g., 35‐year‐old Nigerians and a 34‐year‐old Ghanaian) presented with chronic cough or dyspnea. Other cases were incidental findings during unrelated work‐ups (for back pain, diarrhea, cystitis, etc.). In all reports, chest radiography and ECG initially revealed dextrocardia, typically followed by echocardiography and CT for definitive confirmation. Notable comorbidities included Kartagener syndrome (the Ethiopian adolescent), polycystic kidney disease (a South African patient), and other chronic illnesses (e.g., type 2 diabetes in an older patient). These patterns—late adult detection, predominantly respiratory symptoms, and reliance on multimodal imaging—mirror our case's profile: an incidental SIT finding in a middle‐aged adult detected by chest imaging, with classic mirrored anatomy and no additional anomalies (Table S1; [16, 17, 18, 19, 20, 21, 22, 23]).

Long‐term follow‐up should monitor for subclinical PCD emergence (e.g., spirometry for occult airflow obstruction) and iatrogenic risks during emergencies [12, 13, 14, 15]. Patient education is paramount, as many—like the adolescent—underestimate SIT's clinical significance. This case reinforces that incidental SIT discovery necessitates multidisciplinary planning: anatomical awareness for future surgeries, genetic counseling, and anticipatory guidance even in asymptomatic adults.

## 6 Conclusion

Our case highlights an incidentally discovered isolated SIT in a middle‐aged Ethiopian male presenting with an asthma exacerbation. Comprehensive imaging—including chest radiograph, ECG, ultrasound, echocardiography, and CT—confirmed mirror‐image visceral anatomy and excluded coexisting anomalies. Absence of clinical signs, imaging, or laboratory features consistent with PCD or Kartagener syndrome (e.g., neonatal distress, chronic wet cough, bronchiectasis) supported the diagnosis of isolated SIT. Although asymptomatic and carrying an excellent long‐term prognosis, early recognition is crucial to guide appropriate diagnostic interpretation, surgical planning, anesthetic vigilance, and patient education, thereby mitigating iatrogenic risks.

## 7 Learning Points

- Mirror‐image organ reversal in SIT is often incidental and asymptomatic but demands recognition—especially during ECG, imaging, emergency procedures, and surgical planning.
- Multimodal imaging (radiograph, ultrasound, echocardiogram, CT) is essential to map anatomy, exclude congenital heart disease (~5%–10% of cases), visceral anomalies, and confirm isolated SIT.
- PCD and Kartagener syndrome should be systematically ruled out in SIT patients—neonatal distress, daily wet cough, and bronchiectasis strongly indicate PCD, none of which apply in this case.
- Isolated SIT carries an excellent prognosis; in African and other resource‐limited settings, identification may be delayed into adulthood (46–65 years), underscoring the need for clinical vigilance.
- Patient education and multidisciplinary coordination (anesthesia, surgery, genetics) are vital to prevent procedural complications—such as mistaken organ positions or misplaced incisions—and ensure safe future care.

## Author Contributions

**Ragasa Getachew Bayisa:** conceptualization, data curation, formal analysis, funding acquisition, investigation, methodology, resources, software, supervision, validation, visualization, writing – original draft, writing – review and editing. **Lensa Million Baharu:** conceptualization, data curation, investigation, methodology, resources, software, supervision, validation, visualization, writing – original draft, writing – review and editing. **Mohammed Mecha Abafogi:** conceptualization, data curation, resources, software, supervision, validation, visualization, writing – original draft, writing – review and editing. **Yosef Bekele Bonsa:** conceptualization, data curation, resources, software, supervision, validation, visualization, writing – original draft, writing – review and editing. **Mohammed Kedir Shukri:** conceptualization, data curation, resources, software, supervision, validation, visualization, writing – original draft, writing – review and editing. **Tamirat Godebo Woyimo:** conceptualization, data curation, formal analysis, funding acquisition, investigation, methodology, project administration, resources, software, supervision, validation, visualization, writing – original draft, writing – review and editing.

## Disclosure

The patient conveyed relief at finally being informed about his anatomical variation and expressed strong commitment to annual pulmonary monitoring, feeling empowered by the clarity around his condition.

## Ethics Statement

The authors have nothing to report.

## Consent

The authors received the necessary written informed consent form. The patient offered written informed agreement to the publication of the case's details and accompanying images.

## Conflicts of Interest

The authors declare no conflicts of interest.

## Supporting information


**Table S1:** ccr371425‐sup‐0001‐TableS1.doc.

## Data Availability

All the information used to describe the case is within the article.
