# Supplementary material for: Incidental Diagnosis of Situs Inversus Totalis in a 45‐Year‐Old Male Who Presented With Acute Asthma Exacerbation: A Case Report and Brief Literature Review
Source: Clin Case Rep. 2025 Nov 5;13(11):e71425. doi: 10.1002/ccr3.71425 (PMC12588875; doi:10.1002/ccr3.71425)
Supplement: Supplementary file 1 — Table S1: ccr371425‐sup‐0001‐TableS1.doc. [file CCR3-13-e71425-s001.doc]

Supplementary table 1 summarizes all known African-reported cases of situs inversus totalis—from the first in 2003 to the most recent in April 2025

| **References** | **Year** | **Country** | **Patient Age/Sex** | **Clinical Presentation** | **Diagnostic Method** | **Associated Conditions** |
| --- | --- | --- | --- | --- | --- | --- |
| ***Uchenna DI et.al*** | *2012* | *Nigeria* | *69/F* | *Back pain* | *Chest x-ray, ECG, Echocardiography, Abdominopelvic ultrasound* | --- |
| ***Habtamu M et.al*** | 2025 | Ethiopia | 17/M | Pneumonia | Chest x-ray, ECG, Echocardiography, Chest and abdominal CT scan | KS |
| ***Danbauchi*** | 2003 | Nigeria | 35/M | Respiratory symptoms | Chest x-ray, ECG, Echocardiography, Chest and abdominal CT scan | -- |
|  |  |  | 14/M | Cardiac symptoms | “ | -- |
| ***David A*** | 2009 | Nigeria | Adult Male cadaver | ------ | ------ | ---- |
| ***Olusegun A et.al*** | 2016 | South Africa | 35/M | Respiratory symptoms | Chest x-ray, ECG, Echocardiography, Chest and abdominal CT scan, 99m Tc-MIBI SPECT | PCKD |
| **Abdirahman I et.al** | 2024 | Somalia | 52/M | Acute diarrhea | Chest x-ray, ECG, Ultrasound | T2DM |
| **Nana A et.al** | 2022 | Ghana | 34/F | Respiratory symptoms | Chest x-ray, ECG, Barium meal, abdominal ultrasound |  |
| **Emmanuel K et.al** | 2019 | Ghana | 59/M | Cystitis | Chest x-ray, Barium meal, Abdominal ultrasound |  |
| **CT**: Computed Tomography, **ECG**: Electrocardiogram, **F**: Female, **KS**: Kartagener Syndrome, **M**: Male, **PCKD**: Polycystic Kidney Disease, **T2DM**: Type 2 Diabetes Mellitus, **Tc-MIBI SPECT**: Technetium-99m Sestamibi Single-Photon Emission Computed Tomography. | | | | | | |
